# Severe *Ehrlichia chaffeensis* Infection in a Lung Transplant Recipient: A Review of Ehrlichiosis in the Immunocompromised Patient

**DOI:** 10.3201/eid0803.010249

**Published:** 2002-03

**Authors:** Nasia Safdar, Robert B. Love, Dennis G. Maki

**Affiliations:** University of Wisconsin Medical School, Madison, Wisconsin, USA

**Keywords:** ehrlichiosis, thrombotic thrombocytopenia purpura, immunocompromised host

## Abstract

We describe a case of human ehrlichiosis in a lung transplant recipient and review published reports on ehrlichiosis in immunocompromised patients. Despite early therapy with doxycycline, our patient had unusually severe illness with features of thrombotic thrombocytopenic purpura. Of 23 reported cases of ehrlichiosis in immunocompromised patients, organ failure occurred in all patients and 6 (25%) died.

Since the discovery in 1987 of *Ehrlichia* as a cause of tick-borne disease in humans (*1*), ehrlichiosis has been recognized as an increasingly important cause of acute febrile illness (*2*,*3*). The two main pathogenic species are *Ehrlichia chaffeensis*, which causes human monocytic ehrlichiosis (HME), and the as-yet-unnamed agent of human granulocytic ehrlichiosis (HGE) (*4*). A third species, *E. ewingii,* which has been recently described, causes clinical disease indistinguishable from infection caused by *E. chaffeensis* or the agent of human granulocytic ehrlichiosis (*5*).

Delineation of the epidemiology of human ehrlichiosis has greatly enhanced our understanding of this emerging infection. However, information on the manifestations of ehrlichiosis in immunocompromised patients is limited. We report a case of severe monocytic ehrlichiosis in a lung transplant recipient who had pancytopenia, acute renal failure, and encephalopathy. Despite early diagnosis and treatment with doxycycline, his illness progressed and took on features of thrombotic thrombocytopenic purpura (TTP). A review of reported cases of *Ehrlichia* infection in immunocompromised patients shows that the infection is far more severe in this population and is often fatal.

## Case Report

A 38-year-old man with cystic fibrosis had undergone bilateral lung transplantation in 1998 and had been well. In September 2000, he visited a physician with a 3-day history of fever as high as 38.3°C, myalgias, and headache. A resident of Columbia, Missouri, the patient had spent much time outdoors but did not recall tick infestation or recent tick bite. His medications included cyclosporine, mycophenolate, prednisone, diltiazem, trimethoprim-sulfamethoxazole, and valacyclovir.

On physical examination, the patient appeared acutely ill with temperature 38.3° C, blood pressure 140/64, heart rate 110 per minute, and respiratory rate 20 per minute. He was lethargic but could follow commands, and his neurologic exam was unremarkable. Fine bibasilar crackles were present bilaterally, but heart sounds were normal. Examination of the abdomen was negative. Synovitis was not evident, and no cutaneous lesions were found.

The leukoctye count was 3.7x10^9^ per L with 68% neutrophils, hemoglobin was 64 g/L, and platelet count was 23,000/L. Serum creatinine was 4.6 mg/dL, aspartate aminotransferase 420 U/L, alanine aminotransferase 96 U/L, and bilirubin 3.2 mg/dL. International normalized prothrombin time ratio (INR) was 1.4. Examination of a peripheral blood smear showed schistocytes and other microangiopathic changes.

Multiple blood cultures were negative. Cytomegalovirus DNA was not detected in peripheral blood. Noncontrast computed tomography of the brain was normal. Chest radiograph showed bilateral infiltrates.

The patient was treated initially with intravenous piperacillin-tazobactam and vancomycin. Cyclosporine and trimethoprim-sulfamethoxazole were discontinued. The next day, his mental status continued to deteriorate. Lumbar puncture was deferred because of thrombocytopenia. Antibiotic therapy was changed to intravenous meropenem. Four days after admission, the bone marrow was examined because of worsening pancytopenia; intracytoplasmic morulae were seen in monocytoid cells, characteristic of monocytic ehrlichiosis (Figure). Leukocytes in a peripheral blood smear also contained morulae. Intravenous doxycycline was begun for treatment of presumed *Ehrlichia* infection. Whole-blood polymerase chain reaction (PCR) (Viromed, Minneapolis, MN) in the first week of illness was subsequently reported positive for *E. chaffeensis* DNA. Serology by immunofluorescence antibody testing for both *E. equi* and *E. chaffeensis* performed 2 weeks after onset of illness was negative, with titers <1:40.

**Figure:**
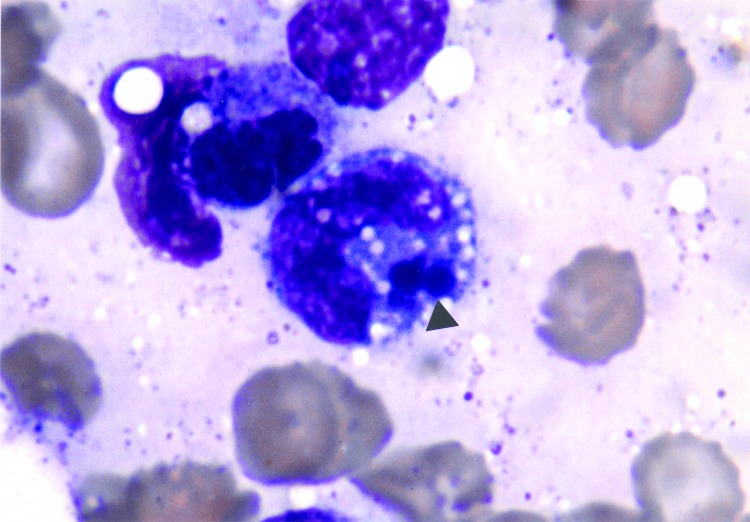
Bone marrow examination (Wright's stain x1000). Intraleukocytic morulae of *Ehrlichia* can be seen (arrow) within monocytoid cells.

Despite treatment with doxycycline, the patient’s confusion, thrombocytopenia, and microangiopathic anemia did not improve, and on the fifth hospital day he was transferred to the University of Wisconsin Hospital and Clinics. Physical examination showed blood pressure 144/94 mmHg, heart rate 77/minute, temperature 36.5ΕC, and respiratory rate 24/minute. Multiple ecchymoses were present on the torso and extremities. Neurologic examination was nonfocal. There were coarse bibasilar crackles in the lungs bilaterally. Examination of the heart and abdomen was unremarkable.

Leukocytes were 2.9 x 10^9^/L, hemoglobin 86 g/L, and platelets 30,000/L. Serum creatinine was 6.2 mg/dL (548 mol/L), total bilirubin 2.0 mg/dL, aspartate aminotransferase 105 U/L, and alanine aminotransferase 55 U/L. INR was 1.1, and the activated partial thromboplastin time was 26 seconds. A peripheral blood smear showed numerous fragmented red blood cells. Chest radiograph showed persistence of bilateral infiltrates.

The patient’s fever resolved 2 days after doxcycline was started; however, oliguric renal failure necessitated hemodialysis. Hematologic studies showed progressive microangiopathic anemia and thrombocytopenia with a normal INR, suggestive of TTP, presumably secondary to *Ehrlichia* infection. Daily plasmaphereses were begun and continued for 8 weeks. Gradually the hematologic abnormalities and renal function improved, and the patient’s mental status returned to normal. Doxycycline was given for 2 weeks.

The patient ultimately made a full recovery with no apparent sequelae. Cyclosporine was not resumed, and he was maintained on sirolimus and prednisone to prevent transplant rejection. No rejection occurred, despite a reduction in immunosuppressive therapy during the treatment of *Ehrlichia* infection.

## Review of Published Reports

Ehrlichiosis is a zoonotic illness caused by *Ehrlichia* species, which are pleomorphic, intracellular, rickettsia-like organisms (*2*–*4*). The clinical spectrum of ehrlichiois varies from a mild, influenzalike illness to a fulminant sepsis syndrome, but in most patients is self-limiting and not fatal. Death rates of documented ehrlichial infection in large, unselected series have been 1% to 8% (*3*,*6*–*8*). This low rate contrasts sharply with the high death rate of ehrliochiosis in immunocompromised patients (Table).

**Table:** Reports of immunocompromised patients with *Ehrlichia* infection

Immunocompromised state	Age	Form of ehrlichiosis	Clinical features	Outcome	Reference
Rheumatoid arthritis, on methotrexate	49	*Ehrlichia ewingii*	Fever, headache	Survived	*5*
Emphysema, on prednisone	65	*E. ewingii*	Fever, headache	Survived	*5*
Renal transplant	11	*E. ewingii*	Lyphadenopathy, fever	Survived	*5*
Liver transplant	47	HME^a^	Multiorgan failure	Survived	*9*
Leukemia	6	HME	Hepatitis, pancytopenia, rash, renal failure	Survived	*10*
Asplenia	71	HGE	Fever, neurologic dysfunction	Survived	*11*
Asplenia	30	HGE	Rash, fever	Survived	*11*
HIV infection (CD4 45/mm^3^)	33	HME	Cardiomyopathy, heart failure	Survived	*12*
Renal transplant	35	HME	Rash, pancytopenia, renal failure	Survived	*13*
HIV infection (CD4 NS)	38	HME	Multiorgan failure	Died	*14*
Sickle beta-thalassemia	NS	HME	Respiratory failure	Survived	*15*
Renal transplant	NS	HME	NS	Survived	*15*
HIV infection (CD4 164/mm^3^)	52	HME	Hepatitis, thrombocytopenia	Died	*16*
Renal transplant	67	HGE	Pancytopenia, renal failure, hepatitis	Survived	*17*
HIV infection CD4 (18/mm^3^)	36	HME	Hepatitis, renal failure	Died	*18*
Liver transplant	51	HME	Pancytopenia, shock	Survived	*19*
Asplenia, chronic lymphocytic leukemia, on steroids	80	HGE	Multiorgan failure	Died	*7*
HIV infection (CD4 199/mm^3^)	37	HME	fever, pancytopenia, toxic-shock-like illness	Survived	*20*
Splenectomy	46	HME	Pancreatitis, shock, encephalitis	Survived	*20*
HIV infection (CD4 64/mm^3^)	41	HME	Pancytopenia, pulmonary hemorrhage	Died	*21*
Asplenia	67	*E. canis*	Renal failure respiratory failure, encephalitis	Died	*8*
Crohn disease requiring prednisone	57	*E. canis*	Pancytopenia, hepatitis	Survived	*22*
Bilateral lung transplant	38	HME	Pancytopenia, renal failure, TTP-like illness	Survived	Current case

^a^HME = human monocytic ehrlichiosis; HGE = human granulocytic ehrlichiosis; NS = not specified

Cellular immunity represents the most important host defense against rickettsial infection (*23*). Acute-phase sera of patients with HGE contain elevated levels of interferon gamma, which is associated with the clearance of *Ehrlichia* from peripheral blood (*24*). In a mouse model of ehrlichiosis, immunocompromised mice have persistent infection, and most eventually die (*25*). Impairment of cellular immunity, whether from immunosuppresssive therapies or underlying disease, retards recovery, leading to more severe disease and higher death rates.

The population of immunocompromised patients is large and growing; many have asplenia or solid organ or bone marrow transplants (*26*). An analysis of the published reports of ehrlichial infection shows that the disease in immunocompromised patients is far more severe and prolonged and more likely to be fatal (Table) (*5*,*7*–*22*). Virtually all these patients had signs of organ dysfunction, including pancytopenia (40%), renal failure (24%), respiratory distress (14%), shock (28%), and neurologic dysfunction (18%). Six (25%) of 23 patients died; 4 of the 6 deaths were in HIV-infected patients. Two patients died within 24 hours after coming to medical attention, despite initiation of appropriate antimicrobial therapy; in the third, the diagnosis was not considered until late in the hospital course; and in the fourth, the diagnosis was made postmortem. Two deaths occurred in asplenic patients; in both, *Ehrlichia* infection was not suspected until 1 week after onset of illness.

In a recent series of ehrlichial infection in 21 HIV-infected patients, 6 of which are included in our review, Paddock et al. reported a high frequency (71%) of moderate to severe disease in HIV-infected patients, particularly with *E*. *chaffeensis* (*27*). Low CD4 counts were associated with a poor outcome.

## Discussion

To our knowledge, this is the first reported case of acute ehrlichiosis in a lung transplant recipient. Our patient had laboratory features typical of *Ehrlichia* infection (thrombocytopenia, leukopenia, and transaminase elevation). However, he also had microangiopathic anemia, renal failure, and neurologic dysfunction characteristic of TTP. Ehrlichiosis with features of TTP has been described in two reports (*28*,*29*), one case each of HME and HGE. Both cases were in immunocompetent persons: one was treated with doxycycline and plasmapheresis; in the other, the diagnosis was made postmortem.

Our patient's multiorgan failure and hematologic aberrations persisted, despite doxycycline therapy, until he underwent plasmapheresis. He was receiving cyclosporine, which is a well-known cause of a rare hemolytic uremic syndrome-TTP-like condition that does not respond to plasmapheresis and nearly always proves fatal (*30*). That our patient’s TTP-like illness coincided with *Ehrlichia* infection and responded to doxycycline and plasmapheresis makes it most likely that it was a consequence of acute ehrlichiosis, not cyclosporine.

Neurologic manifestations, ranging from confusion to frank meningitis, have been reported in up to 20% of patients with erhlichiosis (*31*). Our patient had obtundation and delirium that persisted after doxycycline therapy was initiated and his fever had resolved. The presence of headache and confusion in conjunction with pancytopenia and transaminase elevation should raise suspicion of *Ehrlichia* infection, especially if the patient has had potential tick exposures.

The diagnosis of ehrlichiosis is often delayed because of its nonspecific clinical and laboratory manifestations. In the immunocompromised person, the search for opportunistic infections may further preclude consideration of *Ehrlichia* infection. The empiric antimicrobial regimens used in immunocompromised patients for suspected cryptogenic bacterial and fungal sepsis rarely include a drug or drugs effective against *Ehrlichia*. PCR to detect *Ehrlichia* DNA is invaluable for the diagnosis and has >90% sensitivity and even better specificity (*32*). This technique is particularly useful in the immunocompromised host in whom rapid diagnosis is of utmost importance. Peripheral blood and bone marrow examinations show intracellular morulae in HME in only 1% to 5% of cases and cannot be relied on diagnostically, unless positive. Serologic testing does not allow rapid diagnosis and may be negative in the immunocompromised patient (*21*), as was the case with our patient.

The diagnosis of ehrlichiosis should be considered in any patient with fever, transaminase elevations, and new-onset thrombocytopenia or leukopenia who has had potential tick exposures in an endemic area. In the immunocompromised host, clinical manifestations are more severe and can include neurologic deterioration, multiorgan failure, and even a TTP-like illness. Response to appropriate therapy with doxycycline may be delayed. A high index of suspicion, the use of PCR for confirmatory diagnosis and early empiric therapy can be life-saving.
